# Pneumomediastinum as a complication of SABR for lung metastases

**DOI:** 10.1186/s13014-015-0330-y

**Published:** 2015-01-23

**Authors:** María Esperanza Rodríguez-Ruiz, Estefanía Arévalo, Ignacio Gil-Bazo, Alicia Olarte García, German Valtueña, Marta Moreno-Jiménez, Leire Arbea-Moreno, Javier Aristu

**Affiliations:** Department of Radiation Oncology, Clínica Universidad de Navarre, Avda. Pío XII s/n, 31008 Pamplona, Navarre Spain; Department of Medical Oncology, Clínica Universidad de Navarre, Avda. Pío XII s/n, 31008 Pamplona, Navarre Spain

**Keywords:** Oligometastatic lung disease, Pneumomediastinum, SABR (stereotactic ablative body radiation), SBRT (stereotactic beam radiation therapy)

## Abstract

**Background:**

Stereotactic ablative body radiation (SABR) is a novel and sophisticated radiation modality that involves the irradiation of extracranial tumors through precise and very high doses in patients with oligometastatic lung disease and primary lung tumors.

**Case presentation:**

A 52-year-old female with subclinical idiopathic interstitial lung disease (ILD) and oligometastatic lung disease from squamous urethral cancer who was treated with SABR for a metastatic lesion located in the right lower pulmonary lobe. The patient received a hypo-fractionated course of SABR. A 3D-conformal multifield technique was used with six coplanar and one non-coplanar statics beams. A 48 Gy total dose in three fractions over six days was prescribed to the 95% of the PTV. The presence of idiopathic ILD and other identifiable underlying lung conditions were not taken into account as a constraint to prescribe a different than standard total dose or fractionation schedule. Six months after the SABR treatment, a CT-scan showed the presence of a pneumomediastinum with air outside the bronchial tree and within the subcutaneous tissue without co-existing pneumothorax. To our knowledge, this is the first case of pneumomediastinum appearing 6 months after SABR treatment for a lung metastasis located in the perihiliar/central tumors region as defined by the RTOG protocols as the proximal bronchial tree.

**Conclusion:**

Radiation oncologist should be aware of the potential risk of severe lung toxicity caused by SABR in patients with ILD, especially when chemotherapy-induced pulmonary toxicity is administered in a short time interval.

## Background

Stereotactic ablative body radiation (SABR) is a novel and sophisticated radiation modality that involves the irradiation of extracranial tumors using precise and very high doses of radiation delivered in one to five fractions. This modality has been used in the treatment of non-metastatic primary cancer and also in the case of oligometastases [1]. Phase I/II studies data from patients with oligometastatic lung disease who were treated with SABR or SBRT (stereotactic body radiation therapy), seem to be very promising with high local control rates and long-term survivors in properly selected patients [2,3].

SABR for lung metastases can produce several lung toxicities, such as airways collapse, atelectasis, symptomatic pneumonitis, fibrosis and pathological vertebral fracture [4]. The incidence of symptomatic radiation pneumonitis or grade 3–4 pneumonitis is low, in the range of 0-5% [5]. The risk of pneumonitis is associated with the type of chemotherapy regimen previously administrated, dosimetric parameters, and patient’s age. Non-pulmonary adverse effects, such as esophagitis, esophageal stenosis, massive hemoptysis, pleural effusion and pericardial effusion have been described in patients treated with SABR. The development of these adverse events depend on the location of the tumor and the dose regimen used. A phase II study conducted at Indiana University observed excessive toxicities in 70 patients with central and perihiliar lung tumors treated with high doses of radiation delivered during SBRT (60–66 Gy in three fractions) [3]. In their cohort, twenty percent of patients experienced severe toxicity, including decline in pulmonary function tests, pleural effusion, and pneumonia. Authors informed of 6 treatment-related deaths, the majority of which were due to pneumonia. Moreover, patients with central tumors showed an 11-fold increased risk of presenting a severe toxicity compared to patients with peripheral lesions.

To our knowledge, we report the first case of pneumomediastinum appearing 6 months after treatment with SABR for a solitary lung metastasis located in the perihiliar/central tumors region defined by the RTOG protocols as the proximal bronchial tree.

## Case presentation

A 52-year-old woman with idiopathic interstitial lung disease was referred to our department in March 2010 for a second opinion regarding a metastatic urethral carcinoma. Her history began one year ago when she noticed a node in the urethral meatus by self-palpation associated with perineal pain. Magnetic Resonance (MR) images demonstrated a 2 x 1.5 cm urethral nodule with urethral thickening including the trigone and a single iliac lymph node of 1.2 cm of diameter. A cystoscopy with biopsy revealed a well differentiated infiltrating epidermoid urethral cancer with positive surgical margins. Staging CT-scans were performed showing no distant metastatic disease. On July 2010, the patient underwent anterior pelvic exenteration, urostomy with ileal bladder reconstruction and vaginal reconstruction with a transverse rectus abdominis myocutaneous (TRAM) flap.

The disease was followed until November 2010, when the physical examination demonstrated the presence of a nodule of 3 cm in the right subvaginal space and bilateral metastatic inguinal lymph nodes. The positron emission tomography/computed tomography (PET-CT) revealed tumor recurrence near of the neovagina, the presence of bilateral inguinal lymph nodes and a single lung metastases. A fine-needle aspiration biopsy was obtained from inguinal lymph nodes and the pathologic study confirmed lymph node metastasis of squamous-cell carcinoma of the urethra. The patient was initially treated with six cycles of chemotherapy using paclitaxel 175 mg/m^2^ on day 1, cisplatin 75 mg/m^2^ on day 1 and capecitabine 750 mg/m^2^ on days 1–15 every 21 days. Complete clinical response was observed after six cycles of chemotherapy.

Between March and May 2011, a consolidation hypofractionated course of IMRT (intensity-modulated radiation therapy) was delivered using the step-and-shoot technique. The IMRT dose administrated was 62 Gy prescribed to the 95% of the clinical target volume (CTV = urethra and inguino-pelvic lymph node (LN) in 25 fractions, 2.48 Gy/fraction. Three cycles of chemotherapy with paclitaxel 50 mg/m^2^ and Cisplatin 30 mg/m^2^ on a weekly regimen were concomitantly given throughout the whole course of IMRT. IMRT was followed by a short course of high-dose-rate (HDR) intravaginal brachytherapy. A dose of 4.75 Gy was prescribed at minimum target dose (MTD), over three consecutive days with a total dose of 19 Gy.

**Figure 1:**
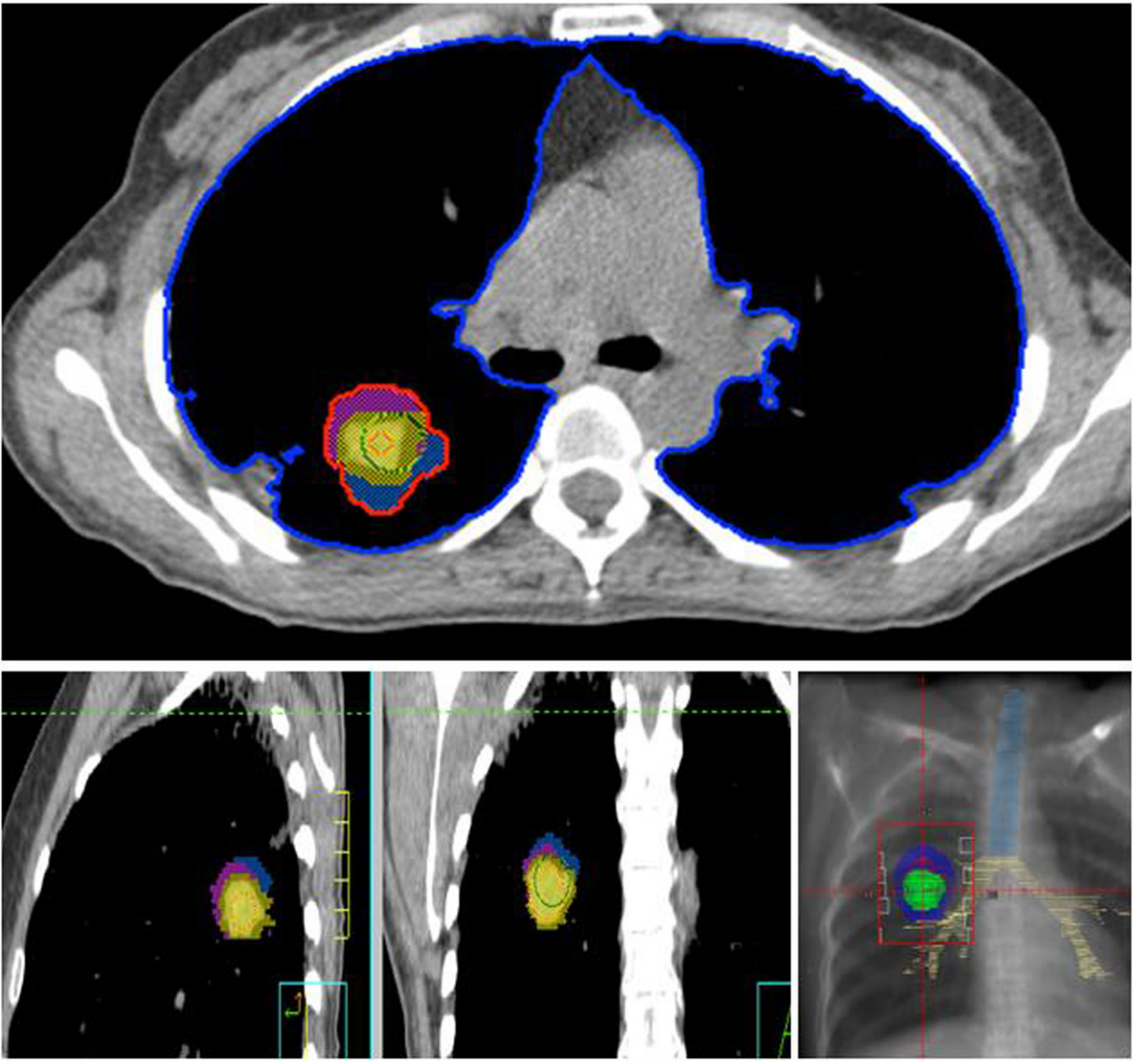
**A combination set of three CT scans obtained during free breathing (pink), deep inspiration (yellow) and deep expiration (blue) were used to generate an internal target volume (ITV) that accounts for respiratory motion (red).** The blue dots outline the lung structure and the red dots indicate the radiation target. Digital Reconstructed Radiography (DRR) show the trachea (blue) and bronchial tree (yellow) structure and the targets volumenes (the lung nodule (green), and the PTV (dark blue) included the ITV (red) with a symmetric 0.5 cm margin.

**Figure 2:**
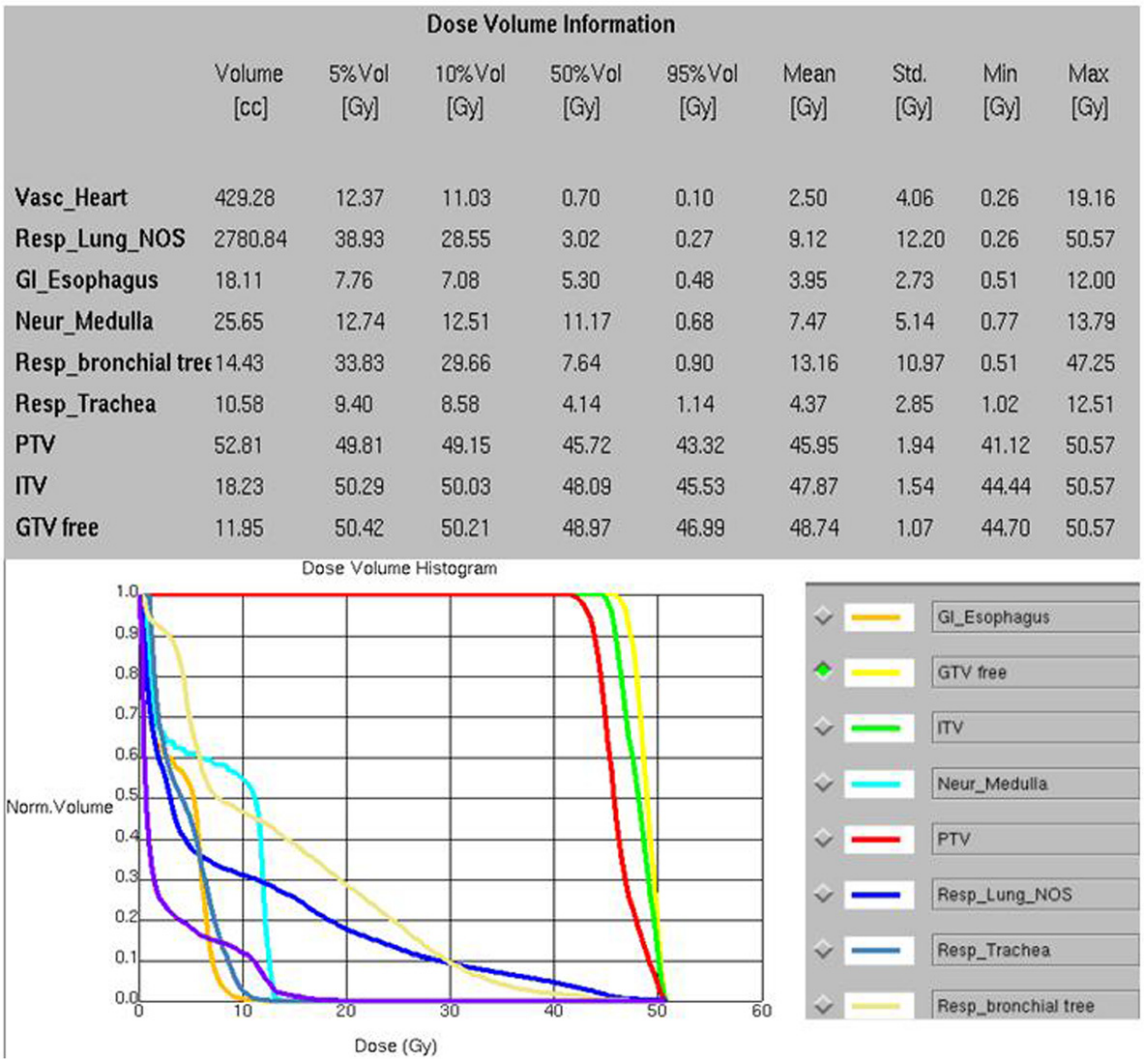
**Dose volume histograms showing dosimetric parameters analyzed including targets and organs at risk.**

The patient was followed until June 2011, when the image studies confirmed a single metastatic lesion on the right lower lung lobe as the unique location of metastatic disease. With the diagnosis of disease progression with a single lung metastatic lesion measuring 30 X 26 mm, a hypofractionated course of SABR was delivered to the metastatic lung tumor. She was placed in supine position and was immobilized using an alpha cradle device to improve patient position reproducibility during daily treatments. Respiratory tumor movement was observed under fluoroscopy. A planning CT scan was performed with a combination of three sets of CT scans obtained during free breathing, deep inspiration and deep expiration to generate an internal target volume (ITV) that accounts for respiratory motion. The gross tumor volume (GTV) was the lung nodule, and the PTV included the ITV with a symmetric 0.5 cm margin (Figure 1). “Organs at risk (OARS) were delimited and constraints were established based on recommendations used in recent RTOG SABR trials (RTOG 0618, 0813 and 0915) and NCCN guidelines (Figure 2). This guidelines concluded that to minimize toxicity when treating lesions in close proximity to the bronchial tree, the Dmax of the trachea and proximal bronqui should be < 30 Gy. In this patient, the Dmax was 12.51 Gy (10.5 cc) and 30,9 Gy (1,69 cc), respectively”.

**Figure 3:**
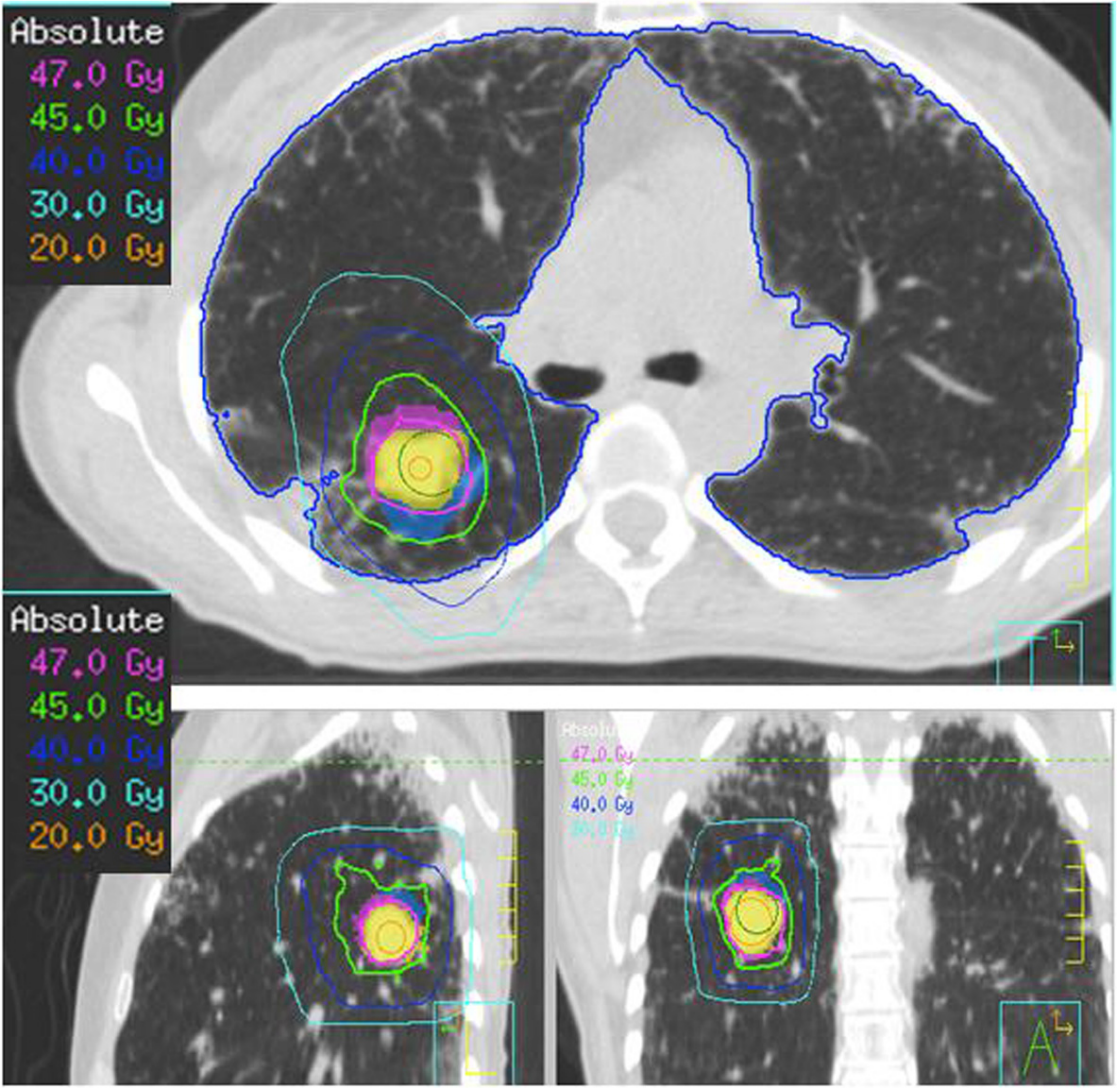
**Dose distribution in the treatment course: Radiotherapy treatment planning with high conformity.** Pink, green and dark blue and light blue areas represent 47 Gy, 45 Gy, 40 Gy and 30 Gy, respectively. The blue dots outline the lung structure and the sky-blue dots indicate the radiation target.

A 3D-conformal multi-field technique was selected with six coplanar and one non-coplanar statics beams. SABR was delivered by a linear accelerator (Oncor, Siemens, Palo Alto, California, USA) with 6-MV photons. A total dose of 48 Gy in three fractions over a six days period was prescribed to the 95% of the PTV (Figure 3). Patient positioning and isocenter verification were checked using cone beam CT. The patient had an excellent inmediate tolerance to the SABR treatment.

## Results

**Figure 4:**
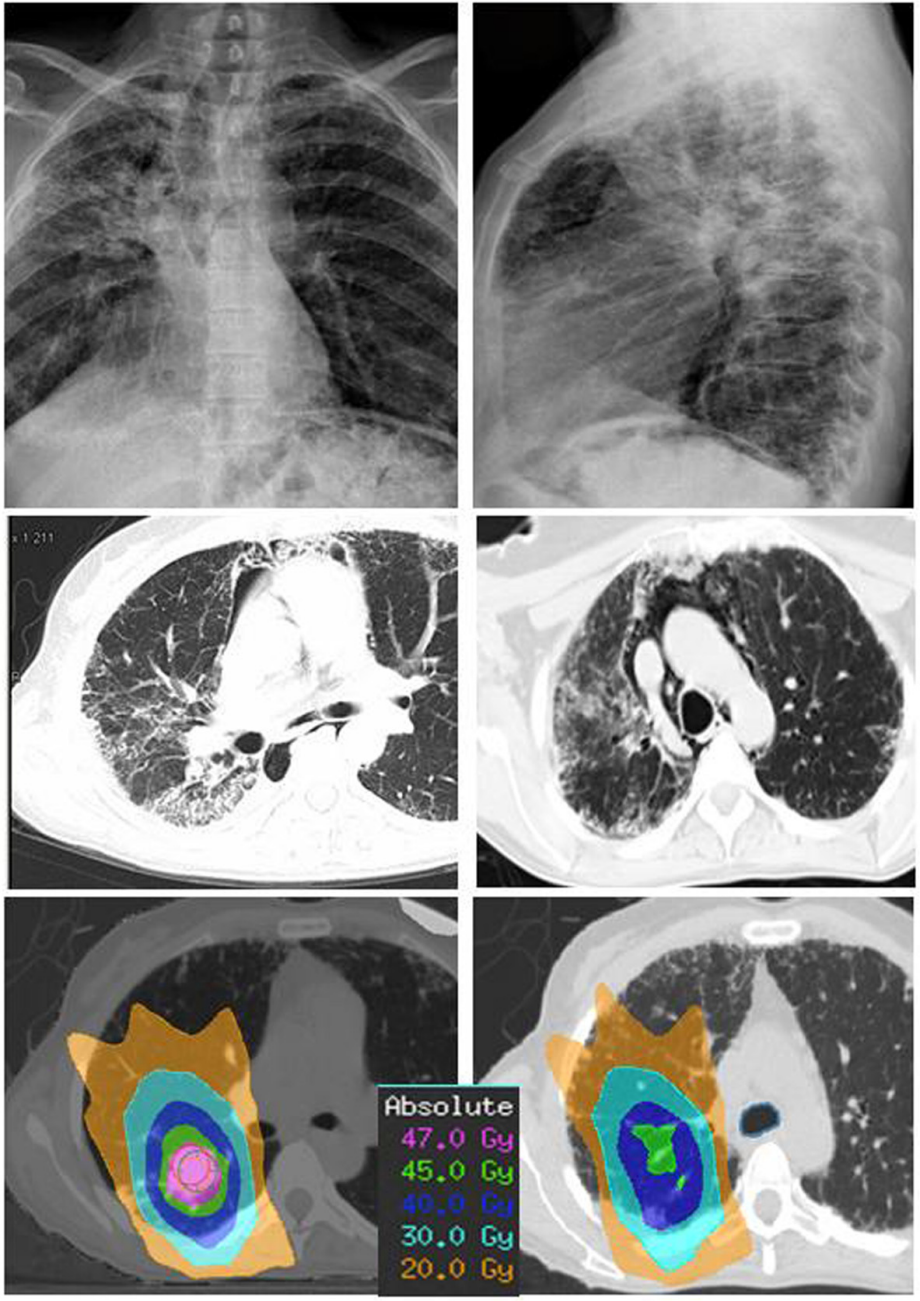
**AP and lateral view of Chest X-ray show the presence of free air in the mediastinal cavity.** Transverse Chest computed tomography (CT) after SBRT shows ground-glass opacities, bleeds confined to the right upper lung, and diffuse ground-glass attenuation, bleeds in the marginal areas, airspace consolidation, free air in the mediastinal cavity and fibrosis in the bilateral upper and lower lung fields. Transverse chest CT view of radiation dose distribution in the treatment course**.** Pink, green and dark blue, light blue and orange areas represent 47 Gy, 45 Gy, 40 Gy, 30 Gy and 20 Gy, respectively.

The patient continued a regular follow-up program including clinical history, physical examination, complete blood count, liver and renal function tests and thoracic, abdominal, and pelvic CT scans every 3 months. Six months after the SABR procedure she referred cervical pain and progressive respiratory deterioration with cough, expectoration and dyspnea. Chest X-ray showed diffuse reticular interstitial lesions in both lungs. The bacteriological and fungal culture of blood and sputum were negative. The chest CT scan continued to show complete response of the metastatic lung disease with signs of radiation pneumonitis and a reticular pattern with shaped diffuse irregular interlobular thickness and little honeycombing pattern of the right lung in accordance with her known chronic lung fibrosis. Surprisingly, the CT scan also illustrated a new image of a pneumomediastinum with air outside the bronchial tree with the presence of diffuse subcutaneous air without co-existing pneumothorax. The patient was diagnosed of radiation pneumonitis and pneumomediastinum in an idiopathic pulmonary fibrosis setting (Figure 4). A bronchoscopy was done to rule out the presence of an airway leak showing no pathological findings at the procedure. clarithromycin 500 mg/12 h, steroid therapy with dexametasone 4 mg/8 h and, E-vitamin were administered for inflammatory lung disease. Two weeks after treatment initiation, the patient remained clinically stabilized and radiological response of pneumomediastinum but a MRI of the spinal cord was performed due to the appearance of moderate neck pain. MRI showed a massive vertebral metastatic (C3) lesion that was treated with palliative radiation therapy (35 Gy in 14 fractions). The patient continued a regular follow-up program and remained asymptomatic but she was diagnosed of multiple bilateral lung metastases in a CT scan and multiple spinal cord metastases in MR images. A second line of chemotherapy was initiated with carboplatin (AUC 3) and gemcitabine 1300 mg/m^2^ every two weeks. Despite this treatment the patient had a progressive clinical and radiological worsening and she died on November 6, 2011. The family refused autopsy.

## Discussion

The development of novel and sophisticated irradiation techniques such as SABR represents a progress in planning and delivering external beam radiotherapy. SABR delivers large doses of radiation in few fractions, and it has been demonstrated to be a safe, effective and promise ablative technique for the treatment of selected patients with lung metastases. Patients diagnosed of oligometastatic lung disease who are considered inoperable or patients that are not candidates for surgical treatment, SABR emerges as a practical option. The reported 2-year overall survival ranges from 67% to 100% and the 2-year local control rate has been established between 92% and 95% [6].

We present the first case of SABR-induced pneumomediastinum in a patient with a solitry lung metastasis and subclinical interstitial lung disease (ILD). A total dose of 48 Gy in three fractions over six days was administered to the 95% of the PTV (Figures 3-4). The PTV, which includes setup uncertainty and residual target motion was designed from the GTV by enlarging the volume 0.5 cm in the axial plane and 1.0 cm in the cranial-caudal plane in all directions. In this particular case, we discard abdominal compression to reduce respiratory motion because comparing free breathing and abdominal compression there was no differences in tumor motion.

Radiation pneumonitis (RP) is one of the most significant complications for patients treated with SBRT [7] and fatal radiation toxicity has been reported in patients with centrally-located stage I lung cancer [3]. Timmerman et al. described six SBRT-related deaths in patients with inoperable stage I lung cancer treated with 60–66 Gy in three fractions during 1–2 weeks. The causes of death were pneumonia, pericardial effusion and massive hemoptysis.

Subclinical ILD was not found to be a significant factor for grade 2–5 RP or clinical outcomes in a recent Japanese study where 100 patients with 124 lung tumors were treated with SABR. Patients with subclinical (untreated and oxygen-free) ILD were treated with SABR (48 Gy in four fractions), while those with clinical ILD (post- or under treatment) were not. However, a significant finding was the presence of uncommon extensive RP in patients with subclinical ILD [8]. Our patient was previously diagnosed of subclinical ILD and she presented pneumomediastinum confirmed by the presence of free air in the mediastinal structures 6 months after SBRT. Another risk factor in this patient associated with severe toxicity is the administration of very high doses of radiation in three fractions (16 Gy x 3) in a lesion located in the perihiliar region. In the other hand, we think that the presence of idiopathic ILD and other lung disease should be taken into account as a more restrictive constraint trying to achieve a BED > =100 Gy. These selected patients can be treated with 4 to 10 fractions risk-adapted SABR regimens.

To our knowledge, pneumomediastinum has not been associated as a complication related to SABR of a primary lung cancer or lung metastasis. Pneumomediastinum was described in the early 19th century by the pathologist R. T. Läennec, who first observed this condition in trauma patients [9]. Primary or spontaneous pneumomediastinum (SPM) is caused by increased pressure gradient between the alveoli and pulmonary interstitium. SPM represents approximately 1% of all cases of pneumomediastinum (PM) and is generally a benign process that mainly affects young people, especially male smokers [10]. Underlying lung conditions as asthma, ILD (loss of integrity of the alveolar-capillary membrane), pneumonia, bullous lung, and radiation therapy for lung cancer have all been associated with SPM [10]. ILD has been identified as the most frequent predisposing factor to PM [11]. Franquet et al. [12] found SPM in 4 patients of 78 patients (5.1%) with idiopathic pulmonary fibrosis. They assessed the presence and distribution of pulmonary parenchymal abnormalities on CT images at the time of detection of pneumomediastinum, and the author observed parenchymal abnormalities such as ground-glass attenuation or honeycombing pattern with a subpleural predominant distribution.

In a study of 34 patients with interstitial pulmonary fibrosis, Fujiwara et al. [13] identified pneumomediastinum on chest CT scans in 5 patients (14.7%). However, they did not assess parenchymal abnormalities at the onset of pneumomediastinum, and assumed that honeycombing pattern and violent cough were predisposing factors for pneumomediastinum.

Our patient had evidence of idiopathic ILD observed several years ago in CT images showing an irregular septal and reticular pattern located in the axial and subpleural interstitium predominantly in the peripheral and basal regions. Reviewing the treatment plan, the treated metastatic lesion was located into the area with higher density of interstitial pattern, the volume receiving at least 20 Gy (V20) was 18% and the mean lung dose was 7.81 Gy (Figure 3-4). These dosimetric parameters are considered in the literature as low risk of pneumonitis after SBRT. To our knowledge, the spontaneous pneumomediastinum and pneumotorax in lung cancer following chemotherapy and radiotherapy may occur due to a variety of mechanisms such as: 1) the rapid tumor lysis and defective tissue repair leading to rupture of a periphereal necrotic pulmonary nodule into the pleural space and/or the bronchus resulting in a bronchopleural fistula and pneumotorax; or 2) the rapid formation of a tumor cavity after radiation therapy with subsequent enlargement and penetration into pleural space. No radiation dose–response effect has been described in the literature, although pneumothorax and pneumomediatinum were not observed in patients who received less than 3000 cGy [14,15]. Chemotherapy also does not appear to correlate with the occurrence of SPT. However, SPT in patients with ILD receiving potentially pneumotoxic chemotherapy, such as chemotherapy-induced interstitial fibrosis and pneumonia, usually SPT is severe, bilateral, and/or recurrent [16].

In addition to ILD, our patient had other lung conditions associated with PM as to have a metastatic cancer in the lung and to receive pneumotoxic chemotherapy.

Pulmonary toxicities due to gemcitabine have been reported. Dyspnea occurs in approximately 25% of patients treated with gemcitabine, whereas serious pulmonary toxicities are much less common, approximately 0.3% of patients present severe pulmonary adverse effects [17]. Gemcitabine can induce radiation recall reactions [18] and the elapsed time required for recall reaction after gemcitabine administration range from 3 days to 8 months [19]. Our patient developed pneumomediastinum 2 weeks after gemcitabine administration and 6 months after SBRT treatment and it is difficult to asses the relative input of gemcitabine in the development of pneumomediastinum but a probable synergistic effect between gemcitabine and SBRT could have been responsible for this severe pulmonary toxicity.

Perhaps, the presence in this patient of multiple predisposing factors with the addition of a precipitating factor such as the delivering of an ablative radiation dose have caused the loss of integrity of the alveolar-capillary membrane and consequently the development of pneumomediastinum.

## Conclusions

We describe a 52-year-old female with oligometastatic lung disease from squamous-cell urethral carcinoma who was treated with SABR for a solitary metastatic lesion located in the right lower pulmonary lobe. The presence of idiopathic ILD and other identifiable underlying lung conditions were not taken into account as a constraint to prescribe a different total dose or fractionation schedule.

Radiation oncologist should be aware of the potential risk of severe lung toxicity caused by SBRT in patients with ILD, especially when pulmonary toxicity-inducible chemotherapy is administered in a short time interval.

## Consent

Written informed consent was obtained from the patient for the publication of this case report and any accompanying images.
